# Ultrasonic particle volume fraction profiling: an evaluation of empirical approaches

**DOI:** 10.1007/s00348-020-03132-0

**Published:** 2021-03-31

**Authors:** Amitosh Dash, Willian Hogendoorn, Christian Poelma

**Affiliations:** Multiphase Systems (Process and Energy) Mechanical, Maritime and Materials Engineering, Delft University of Technology, Mekelweg 2, 2628 CD Delft, The Netherlands

## Abstract

**Abstract:**

We discuss empirical techniques to extract quantitative particle volume fraction profiles in particle-laden flows using an ultrasound transducer. A key step involves probing several uniform suspensions with varying bulk volume fractions from which two key volume fraction dependent calibration parameters are identified: the peak backscatter amplitude (acoustic energy backscattered by the initial layer of the suspension) and the amplitude attenuation rate (rate at which the acoustic energy decays with depth owing to scattering losses). These properties can then be used to reconstruct spatially varying particle volume fraction profiles. Such an empirical approach allows circumventing detailed theoretical models which characterize the interaction between ultrasound and suspensions, which are not universally applicable. We assess the reconstruction techniques via synthetic volume fraction profiles and a known particle-laden suspension immobilized in a gel. While qualitative trends can be easily picked up, the following factors compromise the quantitative accuracy: (1) initial reconstruction errors made in the near-wall regions can propagate and grow along the reconstruction direction, (2) multiple scattering can create artefacts which may affect the reconstruction, and (3) the accuracy of the reconstruction is very sensitive to the goodness of the calibration. Despite these issues, application of the technique to particle-laden pipe flows shows the presence of a core with reduced particle volume fractions in laminar flows, whose prominence reduces as the flow becomes turbulent. This observation is associated with inertia-induced radial migration of particles away from the pipe axis and is observed in flows with bulk volume fractions as high as 0.08. Even transitional flows with low levels of intermittency are not devoid of this depleted core. In conclusion, ultrasonic particle volume fraction profiling can play a key complementary role to ultrasound-based velocimetry in studying the internal features of particle-laden flows.

**Graphic abstract:**

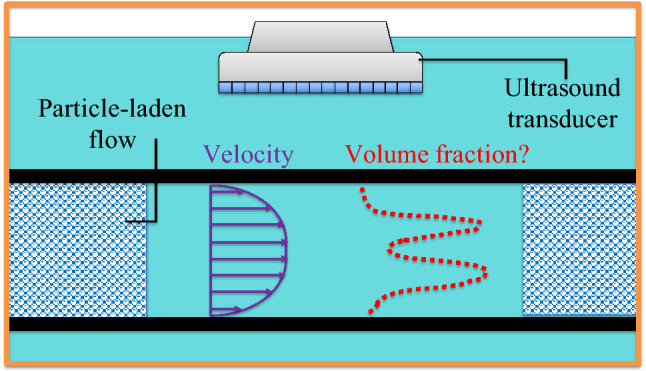

## Introduction and scope

Dispersed multiphase systems are notoriously difficult to access by optical means. The major reason behind this is the presence of numerous scattering interfaces (between the continuous and dispersed phase) which leads to a quick extinction of light, for example in dense sprays (Coghe and Cossali 2012), fluidized beds (van Ommen and Mudde 2008) or bubbly flows (Mudde 2005). This proves problematic for the application of established optical techniques for monitoring purposes.

Due to the complexity in visualizing opaque flows, various measurement techniques for diagnosing such flows have been developed. These techniques have been well summarized and compared in the context of fluidized beds (van Ommen and Mudde 2008), granular mixing (Nadeem and Heindel 2018) as well as dispersed multiphase flows (Poelma 2020). The adoption of acoustical techniques (over X/$\gamma$-ray computed tomography, magnetic resonance imaging, electrical capacitance tomography or radioactive particle tracking) offers several advantages: ease of deployment, lower costs and lower health/safety risks. Moreover, it is completely non-destructive and non-invasive.

Ultrasound based *velocimetry* is relatively well matured (Takeda 2012; Poelma 2017). However, in dispersed multiphase systems, another key quantity of interest is the particle volume fraction (or concentration) profile. Simultaneous measurement of velocity and volume fraction profiles could be instrumental in addressing the micro-structural physics of such flows. The profiling that is discussed in this manuscript should not be confused with commercially available sensors which provide a global/integrated measure of solid volume fractions (Bamberger and Greenwood 2004)

Determination of particle concentration profiles via acoustics is *not* a novel topic and several communities have explored this aspect. We have summarized these efforts in “Appendix A”. One of the communities that has made significant progress on this subject is of sediment transport processes in marine environments (Thorne and Hanes 2002; Hurther et al. 2011; Thorne et al. 2011). In fact, even plug-and-play open source software are being introduced (Fromant et al. 2020). A key feature of their approach is their reliance on meticulously developed theoretical models, while being geared towards specific single-element transducers. The starting point of these models is the scattering induced by a single particle, which is then extended to an ensemble of scatterers. In the end, an equation is obtained between the measured backscattered acoustic signal and the characteristics of the system (distribution of particles, particle sizes, ultrasonic frequency etc.), which is solved to obtain the concentration profile. A brief overview of the interaction between ultrasound and suspensions can be found in “Appendix B”.

The theoretical models often have limitations, especially in terms of maximum volume fractions that can be accurately accommodated (Hunter et al. 2012a). This has led to the development of more generalized approaches over the past decade and can be broadly categorized into: semi-empirical (Hunter et al. 2012b; Rice et al. 2014; Bux et al. 2015; Rice et al. 2015) and empirical (Furlan et al. 2012; Saint-Michel et al. 2017).

A step common to both these generalized approaches involves calibration in a uniform suspension, where the dispersed phase is uniformly distributed across the region of interest. A key difference, however, is how data from calibration tests are used. Semi-empirical approaches integrate the calibration data in the aforementioned rigorous, theoretical scattering models, i.e. the equation between the backscattered acoustic signal and the characteristics of the system. Whereas in empirical methods, the calibration data are used directly to quantify volume fraction profiles in non-uniform suspensions (which is assumed to be composed of multiple, tiny, contiguous regions of uniform suspensions), without the aid of any theoretical scattering model. Empirical approaches offer the advantage of versatility and freedom (for example, the use of linear array ultrasonic transducers or flow configurations with walls) which are not incorporated in the aforementioned rigorous theoretical models.

The focus of the present work is on a detailed assessment of empirical approaches. We outline the general framework as well as address the goodness of the reconstruction. Factors that compromise the quantitative accuracy of the inversion techniques are also highlighted. Our work is performed with linear array transducers (commonly used for medical diagnoses), while the dispersed phase in the studied suspensions have sizes comparable to the ultrasonic wavelength ($\sim$ 0.2 mm). A reader unfamiliar with ultrasound imaging may find “Appendix C” useful, where the basics of the imaging technique and terminology are described. The approaches presented here are best applicable to (semi-)dilute suspensions and for size domains in the order of a few centimetres (laboratory-scale experiments and small/medium-scale industrial flows).

**Fig. 1:**
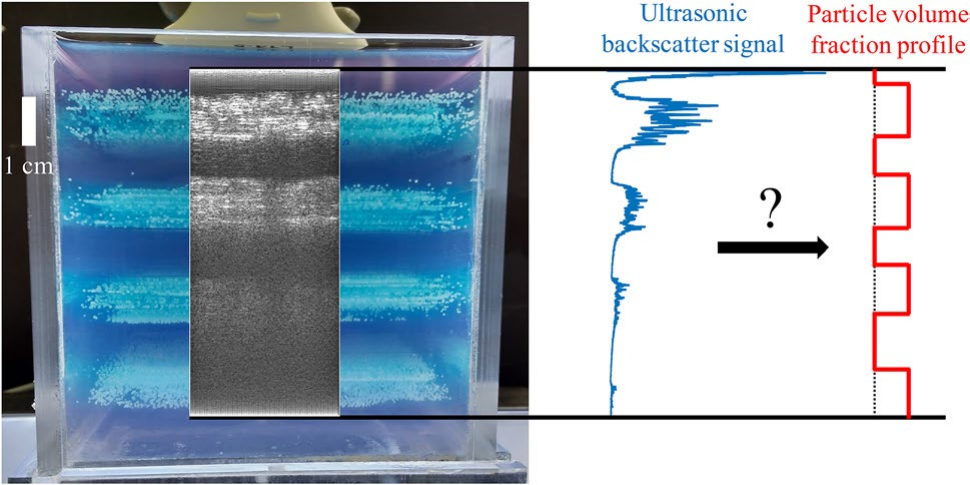
Example of a gelatin model to illustrate the core concept of ultrasound based concentration profiling. Multiple horizontal segments are present, either with or without particles arrested in their position. An ultrasonic image is overlaid

In this manuscript, we describe the key ideas behind an empirical approach, which includes calibration in uniform suspensions (Sect. 2), followed by the application of inversion approaches in profiling non-uniform suspensions (Sect. 3). We explore the applicability of an inversion technique in a model composed of solid particles immobilized in gelatin (Sect. 4). An example of such a model is shown in Fig. 1. These gelatin models help identify a limitation of the technique, namely, multiple scattering (scattering of sound waves between particles, discussed later in more detail). As a case study, we apply the technique to (semi-)dilute particle-laden pipe flows, focusing primarily on radial migration in neutrally buoyant suspensions (Sect. 5). We end by summarizing our key findings and discussing possible directions that can build up on the work presented here (Sect. 6).

## Calibration in uniform suspensions

Before proceeding to the profiling of non-uniform suspensions, a necessary step in an empirical technique would be the characterization of uniform suspensions. Characterization of uniform suspensions can be useful in monitoring the global volume fraction of the system. Unlike the transceiver apparatus (i.e. a single device for transmission and receiving of sound) employed here, uniform suspensions may also be monitored by means of a transmitter and a receiver placed at a fixed separation. The change in speed of sound or attenuation of sound due to the particles in between the two may then be used characterize the suspension and this has been done in previous studies (for example, Stolojanu and Prakash 2001).

**Fig. 2:**
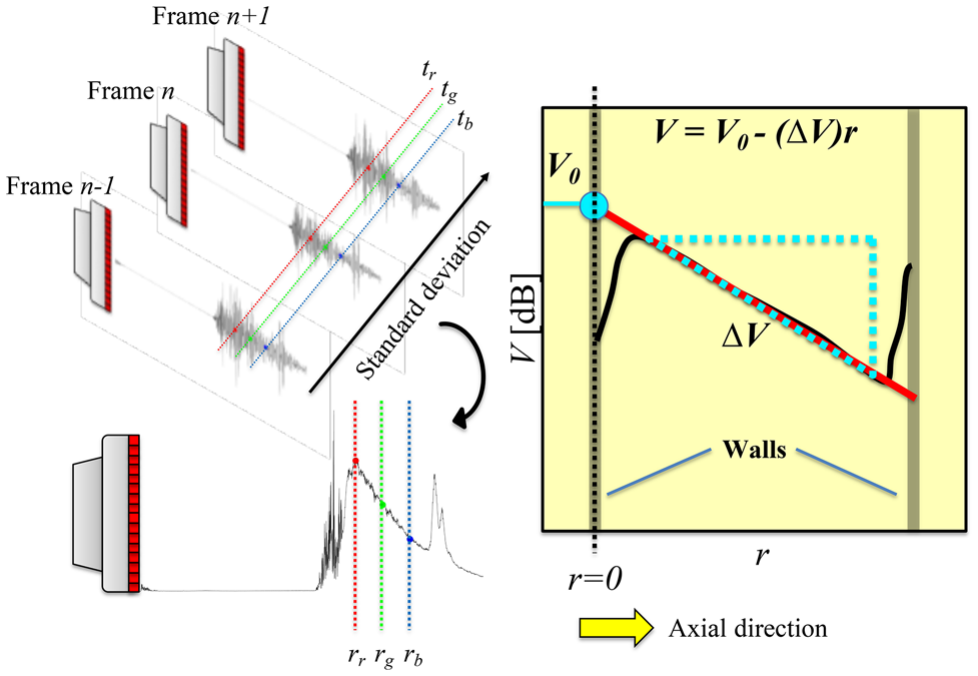
Backscatter amplitude and characterizing a uniform suspension. (Top left) Backscattered signals (post-beamformed RF data) recorded by the transducer in multiple frames. (Bottom left) Derivation of the backscatter amplitude profile. (Right) A simplified schematic of the backscatter amplitude (now in dB) with a linear fit (solid red straight line between walls) through it. This linear fit returns the peak backscatter amplitude (y-intercept) and the attenuation rate (slope) corresponding to the uniform suspension being characterized

For the current experiments, a transceiver is used for measurements, which necessitates the use of different means to characterize the suspensions. The time-of-flight or extinction-of-sound like measurements used in separated transmitter-receiver configurations can not be used. Thus, the technique proposed by Weser et al. (2013a, b, 2014) is adapted and a schematic elucidating this process is shown in Fig. 2.

The procedure is as follows: Every frame in the entire ensemble of measurements is a time series consisting of post-beamformed RF data. We emphasize that ‘frame’ is used in the current context to refer to an individual time series (for example, frame $n-1, n, n+1$ in Fig. 2) and should not be confused with the time instant within a frame at which the backscattered signal is received (for example, $t_r/t_g/t_b$ in Fig. 2). Then, a new series is created out of the data at a fixed time instant across all frames (for example, all the dots of three different colors - red, green and blue - in the top left panel in Fig. 2 form a new series each). The standard deviation of each of these new series is converted to a new time series and thus, a new signal is born. This new signal is referred to as the backscatter amplitude (*V*) henceforth, and it displays an exponential decay with increasing distance, due to attenuation (fixed percentage of sound energy is lost for the depth propagated). In a medium with speed of sound $c_0$, time can be converted to space as $r = 0.5c_0t$. Upon converting the magnitude of the backscatter amplitude into decibels, the exponential decay is converted into a linear decay. Of course, for a flow with extreme temporal fluctuations of the particle volume fraction, the conversion of magnitude to decibels could incur errors for the time-averaged signal due to the inherent non-linearity of the transform. However, this is not an issue for the present study, as the flow is steady in nature. An alternative approach is to use the mean (instead of the standard deviation, respectively) of the A-mode data (instead of the post-beamformed RF data). It must be noted that if the frame is a B-mode image (instead of post-beamformed RF/A-mode data), then the process of converting the backscatter amplitude to decibels is unnecessary. This arises from the fact that the log-compression step in generating the B-mode image is mathematically similar to that of converting a magnitude into decibels.

A straight line may be fitted through this decay profile and two parameters can be obtained: the y-intercept and the slope. For wall-bounded flows, these fits are usually done on the signal corresponding to the central region of the geometry, i.e. away from the walls. This is done so since the backscatter amplitude profile near the walls is less reliable. It has been shown that the y-intercept and the slope show good correspondence with theoretical backscattering coefficients and experimentally measured attenuation respectively (Weser et al. 2013a, b, 2014). Thus, the y-intercept and the slope of this fitted line are referred to as the peak backscatter amplitude ($V_0$) and the attenuation rate ($\varDelta V$) respectively. Note that we consider the attenuation rate as a positive quantity, by fitting the following profile: $V(r) = V_0 - \varDelta V r$.

**Fig. 3:**
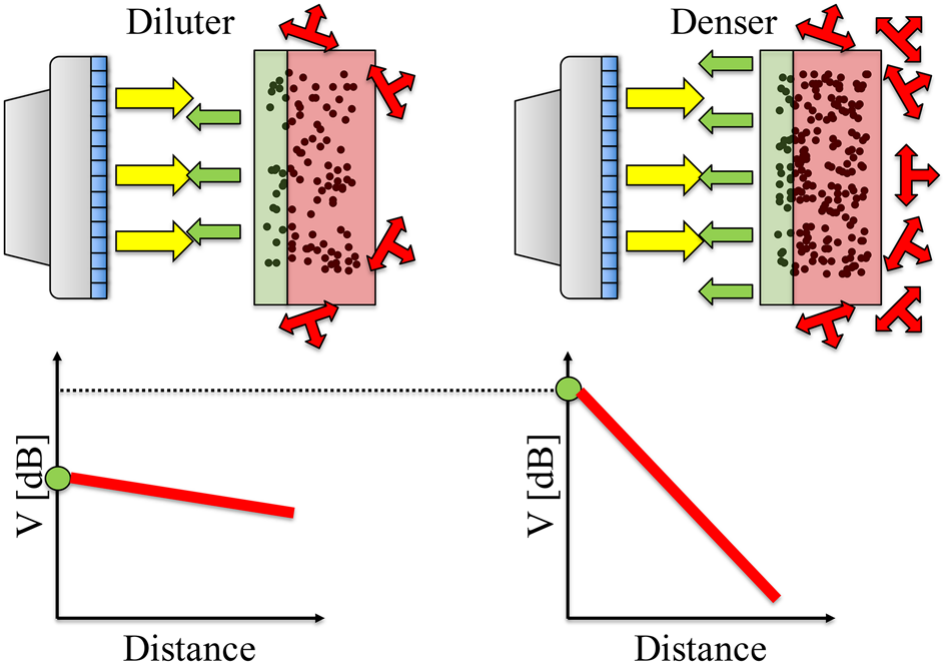
Effect of particle loading on backscatter amplitude. For a fixed insonification (yellow arrows pointing away from transducer), increasing the particle volume fraction in a uniform suspension increases the peak backscatter amplitude (green arrows pointing towards transducer in top panel and green circle in bottom, $V_0$) as the number of scattering interfaces are increased. However, the amount of scattering in the non-backscatter direction increases, leading to a quicker attenuation (groups of three red arrows originating from a node in top panel and solid red straight line in bottom, $\varDelta V$) of the backscatter amplitude

In order to understand the influence of particle volume fraction on the backscatter amplitude characterization, the schematic in Fig. 3 is considered. On the left is a more dilute suspension, while a denser suspension is present on the right. When a fixed amount of acoustic energy is sent into the suspensions by the transducer, the denser suspension initially scatters the sound back to the transducer more intensely, due to the presence of more particles. This leads to higher peak backscatter amplitudes ($V_0$) for denser suspensions. However, as sound propagates through, the denser suspension scatters relatively more sound away from the transducer as well, in comparison to the diluter suspension. This leads to a quicker loss of acoustic energy, i.e. the attenuation rate ($\varDelta V$) is higher as well for denser suspensions.

**Fig. 4:**
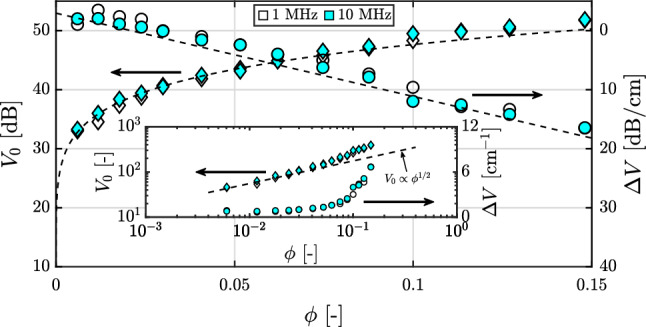
Sample calibration data obtained in a pipe flow with a uniform suspension. Characterization in decibels, but in arbitrary units for inset. Inset in log-log axes for peak backscatter amplitude and semi-log axes for amplitude attenuation rate. Diamonds represent the peak backscatter amplitude and circles represent the amplitude attenuation rate

A sample calibration performed in a pipe flow, as a function of particle volume fraction, is shown in Fig. 4. The calibration was performed in a pipe with an internal diameter of 10.00 mm, while the particle diameters were 530 ± 75 $\mu$m. The suspension is comprised of polystyrene spheres suspended in saline water. A uniform suspension was obtained by having a neutrally buoyant system as well as turbulent flow conditions. For the present experiments, it is expected that the particles possess finite inertia. Fiabane et al. (2012) have shown that neutrally buoyant particles are homogeneously distributed in turbulence, unlike heavy particles, which tend to cluster. Thus, we expect that the calibrations in the present study are not significantly affected by the phenomenon of turbophoresis - the preferential accumulation of inertial particles in regions of low turbulence intensity (Caporaloni et al. 1975; Reeks 1983).

Data acquisition is performed via a SonixTOUCH Research (Ultrasonix/BK Ultrasound) system coupled with a linear array transducer (L14-5/38). The transducer is immersed in a water bath surrounding the pipe, to improve the acoustic coupling. Straight line fits were performed on the backscatter amplitudes corresponding to the central parts of the pipe and the length used for the fit varied from 0.75 - 0.84 cm. Profiles of the peak backscatter amplitude and the attenuation rate from measurements at two central frequencies (1 MHz and 10 MHz, $ka \sim$ 1.1 and 11, respectively; both with one cycle long pulses) are presented. While there is a minor difference between the results at the two central frequencies, the overall trends for the peak backscatter amplitude and the amplitude attenuation rate are in agreement with the expectations illustrated in Fig. 3.

The peak backscatter amplitude in Fig. 4 displays a power-law behaviour with increasing volume fraction and can be characterized by means of an empirical power-law fit, $V_0 = a\phi ^n$ with $n < 1$. This also means that the peak backscatter amplitude is more sensitive to volume fraction changes for lower volume fractions ($\frac{{\rm d}V_0}{{\rm d}\phi } = a n \phi ^{n-1}$). Plotting the peak backscatter amplitude on a log-log scale shows that the theoretical relation, $V_0 \propto \phi ^{1/2}$ (Thorne and Hanes 2002), is valid until $\phi \sim 0.04$ for the present suspension (see inset of Fig. 4). This further strengthens the argument for the need for empirical methods. While not explored here, it has been shown for other suspensions (typically in the long wavelength regime) that the peak backscatter amplitude profile shows non-monotonic behaviour with a maximum attained between volume fractions of 0.10 - 0.30 (Chen and Zagzebski 1996; Wang and Shung 1997; Baddour and Kolios 2005; Franceschini and Guillermin 2012), which would impose a limit to the range of volume fractions that can be studied by the inversion techniques discussed later.

The attenuation shows a very linear behaviour with volume fraction, on the decibel scale, something that was also observed by Weser et al. (2014) for glass beads. The attenuation is characterized by a linear fit $\varDelta V = m\phi + c$. An interesting observation is that the attenuation rate has a negative value at lower volume fractions, i.e. the backscattered energy *increases* with depth. A possible reason for this is the complex three-dimensional shape of the ultrasonic beam, which is commonly characterized by a focal point. The beam typically converges until the focal point and diverges thereafter. Another global property that is a function of particle volume fraction, is the speed of sound. However, for polystyrene suspensions in (saline) water, the speed of sound changes by only about 3% for volume fractions up to 0.15 (Kuster and Toksöz 1974), and its effect can be neglected for the range of volume fractions studied here.

**Fig. 5:**
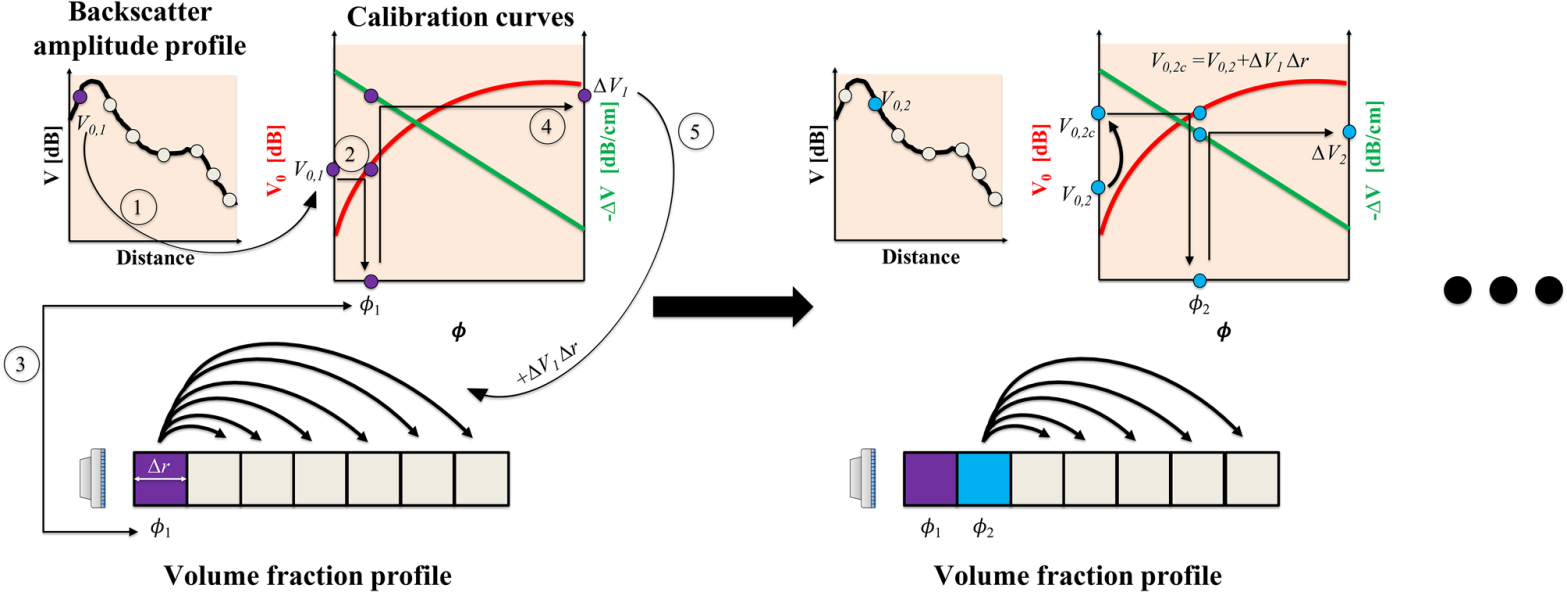
The stepwise reconstruction process for obtaining the particle volume fraction profile. (Left) Estimation of volume fraction in first bin: use $V_{0,1}$ to estimate $\phi _1$ (steps 1-3) which can then be used to find $\varDelta V_1$ (step 4) that will be used in the compensation step (step 5). (Right) Estimation of volume fraction in second bin: estimate $V_{0,2c} = V_{0,2} + \varDelta V_1 \varDelta r$; use $V_{0,2c}$ to estimate $\phi _2$ which can then be used to find $\varDelta V_2$. In the next step (not shown), estimate $V_{0,3c} = V_{0,3} + \varDelta V_2 \varDelta r + \varDelta V_1 \varDelta r$, use $V_{0,3c}$ to estimate $\phi _3$ and so forth

While we have chosen these two functions (power fit for peak backscatter amplitude and linear fit for amplitude attenuation rate) based on our observations, other functions may be chosen as well in order to attain a better fit. This encapsulates the spirit of the empirical nature of the measurement technique. It should be noted that performing ex-situ calibration in a different geometry is not recommended as the flow geometry could also influence the propagation of the sound waves, and thus potentially, the characteristics of the backscatter amplitude profiles. In case a uniform suspension cannot be generated in the system (for example, due to the flow itself), calibration in a 1:1 mock-up could be considered. Moreover, beamform settings should remain unaltered throughout, for a valid comparison. Image settings such as multiple transmit foci is discouraged as this generates artificial discontinuities in the backscatter amplitude profile. The calibration data from Fig. 4 will later be used to create synthetic backscatter amplitude profiles in Sect. 3.3.

## Inversion to obtain particle volume fraction profile in non-uniform suspensions

In this section, we discuss two potential inversion techniques in Sects. 3.1 and 3.2, before applying these to synthetically generated signals in Sect. 3.3.

### Procedure 1: Stepwise reconstruction

The first technique that can be used to invert the backscatter amplitude signal to obtain the particle volume fraction profiles is the stepwise reconstruction technique. As the name suggests, the reconstruction is performed in a stepwise manner, beginning from the transducer and propagating in the initial direction of the acoustic waves. The reconstruction technique can be understood via the schematic in Fig. 5. The time-averaged backscatter amplitude signal, *V*, is divided into a certain number of bins, each with a fixed length, $\varDelta r$. It is assumed that the particle volume fraction is homogeneous within this bin. Each bin has a representative backscatter amplitude value (the bin average).

The reconstruction starts at the bin located closest to the ultrasound transducer. Let us consider the data point closest to the transducer in Fig. 5. For the first bin, the bin-averaged backscatter amplitude, $V_{0,1}$, can be directly compared with the calibration curve of the peak backscatter amplitude (solid red non-linear calibration curve, $V_0$ versus $\phi$) which returns an estimate for the particle volume fraction for that bin ($\phi _1$). However, due to the particles present in this first bin, the backscatter amplitude signal will be attenuated due to scattering. Thus, as a next step, the signal in the following bins are adjusted to compensate for this loss by attenuation (shown by the group of six black arrows originating from the same node). This compensation is the product of the attenuation due to the local particle volume fraction (the solid green linear calibration curve, $\varDelta V$ versus $\phi$) and the bin size, $\varDelta V_1 \varDelta r$.

Following this, when the second bin is considered, the peak backscatter amplitude, $V_{0,2,c} = V_{0,2} + \varDelta V_1 \varDelta r$ is thus appropriate for estimating the local particle volume fraction. Then, the signal attenuation for the second bin, $\varDelta V_2 \varDelta r$, is accounted for in the following bins, i.e. the third bin onwards. This way, the time-averaged particle volume fraction profile, $\phi (r)$, is reconstructed in a stepwise manner.

The stepwise reconstruction process may be expressed with Eq. 1.

1 $$\begin{aligned} V(r) = \underbrace{V_0(\phi (r))}_{1} - \underbrace{\int _0^r \varDelta V(\phi (r')){\rm d}r'}_{2} \end{aligned}$$

While the continuous form of the equation is shown, it can be discretized into bins of finite sizes, in order to solve the inverse problem. This equation can be considered as a simplified, lumped version of the semi-empirical formulations used in determining suspended sediment concentrations, (see Eq. 5 in “Appendix A”). However, the above formulation allows more freedom in characterizing non-dilute suspensions, where scattering of sound may not be trivially expressed with theoretical formulations.

The nature of quantifying particle volume fractions with the stepwise reconstruction bears a strong similarity with the method used in Furlan et al. (2012). However, a key difference is their usage of spectra instead of our definition of backscatter amplitude. While spectra contain much more information, the much simpler backscatter amplitude is sufficient for the current purpose. Both these methods can also be likened to the iterative implicit technique used in the marine sediment transport community (see cluster I, “Appendix A”).

The stepwise reconstruction is also very similar to the approach put forth by Saint-Michel et al. (2017), with one key difference. They acknowledge simplifying the reconstruction by not employing a space-dependent attenuation coefficient. In our terminology, their approach would transform Eq. 1 into $V(r) = V_0(\phi (r)) - r \varDelta V(\phi (r))$, which is free of the integral, allowing them to reconstruct the entire profile in one step.

### Procedure 2: Dual-frequency reconstruction

As can be seen, the integral on the right hand side of Eq. 1 turns the stepwise reconstruction technique into a set of implicit equations, which is susceptible to errors during inversion (Hurther et al. 2011). The implicit nature of the set of equations for the stepwise reconstruction technique (Eq. 1) can prove troublesome in the reconstruction process due to numerical instabilities. A solution that was devised for this issue was the usage of the ultrasonic transducer at two different central frequencies (Hurther et al. 2011). By changing the central frequency of the ultrasonic wave, the relative wavelength would be changed as well. This in turn affects the interaction of the acoustic wave with the particle/suspension.

This inversion technique has so far been used by theoretical (Hurther et al. 2011) as well as semi-empirical approaches (Rice et al. 2014). Here, we rearrange Eq. 1 with the aid of the calibration curve fits, Eq. 2. For each frequency, the coefficients *a*, *n*, *m* and *c* would have different values. The below forms for the curve fits are based on the data in Fig. 4.

2 $$\begin{aligned} V_0=\, & {} a\phi ^n \nonumber \\ \varDelta V=\, & {} m\phi + c \end{aligned}$$

For the above characterization, upon combining Eqs. 1 and 2, followed by a rearrangement of terms, Eq. 3 is obtained.

3 $$\begin{aligned} \frac{V(r)-a\phi ^n(r)+cr}{m} = \underbrace{- \int _0^r \phi (r')dr'}_{\rm{constant}} = -\phi _{\rm{bulk}} \end{aligned}.$$

The term on the right hand side is a constant for a stationary flow, allowing the elimination of the integral term. This is beneficial, as the left hand side of this equation, for the two frequencies may be equated directly, to create a system of explicit equations, as shown in Eq. 4.

4 $$\begin{aligned} \frac{V_1(r)-a_1\phi ^n_1(r)+c_1r}{m_1} = \frac{V_2(r)-a_2\phi ^n_2(r)+c_2r}{m_2} \end{aligned}$$

**Fig. 6:**
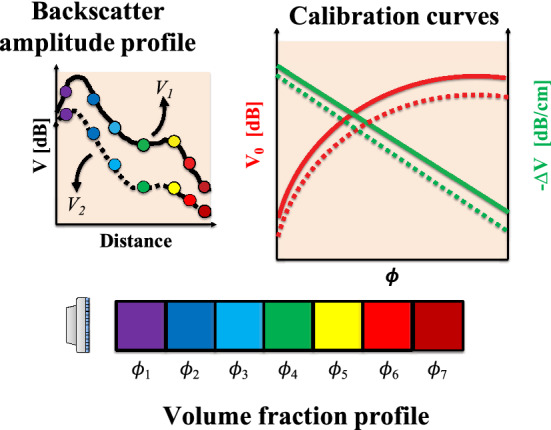
The dual-frequency reconstruction process for obtaining the particle volume fraction profile. (Top left) The backscatter amplitude signal measured at the two frequencies. (Top right) Calibration curves for the two frequencies. (Bottom) Reconstruction of the volume fractions using Eq. 4. The solid and the dashed lines correspond to measurements at the two frequencies

This technique has been shown to have been robuster for sediment transport studies (Thorne et al. 2011) as it is less susceptible to errors arising from numerical instabilities. The process is summarized in Fig. 6 and as it suggests the entire profile can be reconstructed in a single iteration. Such a technique can also be extended to add measurements at a third central frequency of the transducer, which has been shown to further reduce the uncertainty in the estimated concentration profiles (Thorne and Hurther 2014).

In general, it would be desirable to have a calibration function for attenuation where the volume fraction can be trivially separated from the calibration constants. This is not the case when a second order polynomial is used for the amplitude attenuation rate as a function of particle volume fraction. In such a scenario, it would be challenging to formulate an equation resembling Eq. 3, where a constant quantity ($\int _0^r \phi (r'){\rm d}r'$) was isolated.

### Comparison of the two techniques using synthetic profiles

Given the two proposed inversion techniques, a natural question that might arise is which of the two techniques is better. Here, we perform a comparison of the two techniques based on synthetic signals, generated from the experimental data in Fig. 4 and the equation forms in Eq. 2.

**Fig. 7:**
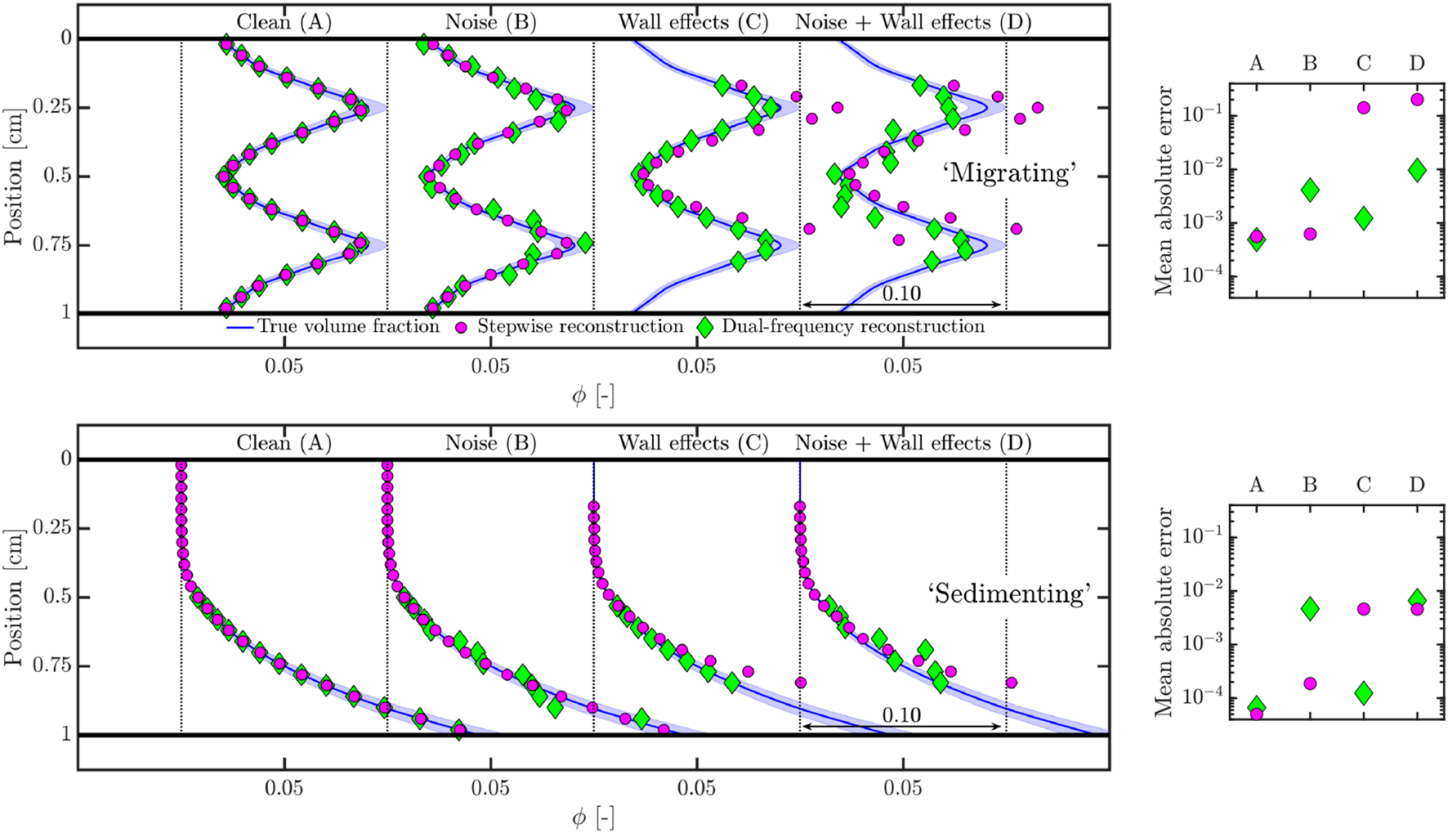
Performance of the two inversion techniques are judged by means of synthetic signals. Panels on the left show the reconstructions while those on the right show the corresponding mean absolute errors of the reconstructions with respect to the true volume fractions. (Top) Profiles mimicking inertial migration of particles. (Bottom) Profiles mimicking transport of heavy particles

A particle volume fraction profile is assumed and the corresponding synthetic signal or ‘forward problem’ is generated with the help of Eq. 1. Coefficients of the curve fits used to characterize the uniform suspensions are summarized in Table 1. This signal is basically a hypothetical backscatter amplitude as a function of distance from the transducer, conforming to the flow/measurement geometry used in the calibration (Fig. 4). Since the calibration coefficients have similar values, it was seen that the separation between the constructed synthetic signals, |*V*_1 MHz_(*r*)−*V*_10 MHz_(*r*)|, is not separated by a large magnitude.

**Table 1:** Coefficients for curve fits used in synthetic signal generation. See Eq. 2 for the curve fitting characteristic equations

Frequency	*a*	*n*	*m*	*c*
1 MHz	65.12	0.1313	129.3	− 3.677
10 MHz	64.70	0.1328	141.1	− 2.971

Two assumed profiles and their corresponding reconstructions are shown in Fig. 7. Two cases are considered: one where the particle volume fraction profile resembles the radial migration of neutrally buoyant particles away from the axis (‘migrating’) and the other where the profile resembles the transport of dispersed particles heavier than the fluid (‘sedimenting’). While the migrating profile has a non-monotonic shape, the sedimenting one is monotonic. Furthermore, for each of the two cases, two undesirable, but unavoidable experimental effects are considered. For example, noise fluctuations are added to the backscatter amplitude signal, which can be expected in any realistic measurement. In the other case, wall effects are considered by corrupting the backscatter amplitude signal near the wall regions. The latter artefact is more relevant to wall-bounded flows, and have also been reported in other works (Wang et al. 2003; Saint-Michel et al. 2017).

First, the reconstruction of a ‘clean’ backscatter amplitude signal (without any noise or wall effects) is considered. It can be seen that both the inversion techniques perform equally well, which is also a verification that the inversion techniques have been implemented properly. The dual-frequency reconstruction technique is unable to estimate a volume fraction for positions between 0 and 0.5 cm. A possible reason for this is that the local volume fractions is very low ($\approx$ 0) which would make Eq. 4 difficult to solve.

Next, white Gaussian noise (signal-to-noise ratio of 15) is added to the backscatter amplitude signals. From the reconstructions, it is evident that the stepwise reconstruction technique performs slightly better than the dual-frequency technique. This happens as the fluctuations induced by noise on the backscatter amplitude signal are of similar order-of-magnitude as the separation of the backscatter amplitude signals recorded at the two frequencies. Nevertheless, both techniques provide satisfactory solutions, certainly at a qualitative level.

Hereafter, wall effects are considered. This is done by altering the backscatter amplitude signals near the wall regions, making these regions less reliable for reconstruction. Basically, the backscatter amplitude profile is manipulated in the near-wall region so that it deviates significantly from what would be expected from Eq. 1. The inversion is then performed only between the regions of 0.15 - 0.85 cm. It is clear that the dual-frequency technique is the robuster one here. This is because of the explicit nature of the system of equations. In the stepwise reconstruction technique, an error is made in the particle volume fraction estimation at the first bin itself (at 0.15 cm). No information of the volume fraction profile is calculable before the first bin (< 0.15 cm), and an assumption needs to be made, i.e. the profile is homogeneous until the first bin. This assumption leads to an initial error which then propagates in an additive manner as the reconstruction moves away from the transducer. The behaviour of the error propagation is different for the two profiles. In both cases, the estimated volume fraction profiles behave in a similar fashion as the actual ones. However, for the ‘sedimenting’ profile, the absolute value of the estimation error is monotonic owing to the monotonic nature of the actual volume fraction profile. The stepwise reconstruction solution beyond a distance of 0.75 cm is not computed as the volume fraction estimate already exceeds 0.15, more than the maximum volume fraction in the calibration. Nevertheless, the stepwise reconstruction technique still provides a good qualitative insight.

Finally, both techniques are compared with Gaussian white noise (signal-to-noise ratio of 5) as well as wall effects. Neither of the two techniques provide acceptable estimates at a quantitative level. This also shows that both artefacts, noise and wall effects, can affect the goodness of the reconstruction.

The discussion above pertained to the extraction of time-averaged profiles. The performance of the two reconstruction techniques are summarized in Table 2. While the effect of noise may be reduced by longer measurements, the quality of the data near the walls can be improved (also while removing the effects of fixed artefacts), with the help of tools such as a temporal high pass filter (Sciacchitano and Scarano 2014).

Sometimes time-averaged profiles may not be sufficient and for dynamic processes, time-resolved monitoring might be desired. The stepwise reconstruction technique would be more appropriate for this purpose. Depending on the relevant time-scales of the flow processes, higher rates of signal acquisition might be required, for example, by means of plane-wave imaging (Tanter and Fink 2014). In order to employ dual-frequency reconstruction, a high rate of signal acquisition in tandem with the possibility to quickly alternate between two central frequencies of the transducer are needed, which is not straightforward. This would also mean that if simultaneous measurements of velocity and particle concentration fields are desired with a single transducer, the stepwise reconstruction technique would be the more appropriate approach, and such measurements have been demonstrated by Saint-Michel et al. (2017).

**Table 2:** A summary comparing the two reconstruction techniques. Comparison based on synthetic signals

Characteristic	Stepwise	Dual-frequency
System of equations	Implicit	Explicit
Handles noise well?	For low noise levels	If $|\varDelta V|>> V_{\rm{noise}}$
Handles wall artefacts?	Initial error occurs which grows in the reconstruction direction	Robust
‘Instantaneous’ concentration profiles	Possible with appropriate hardware	Requires multiple transducers

**Fig. 8:**
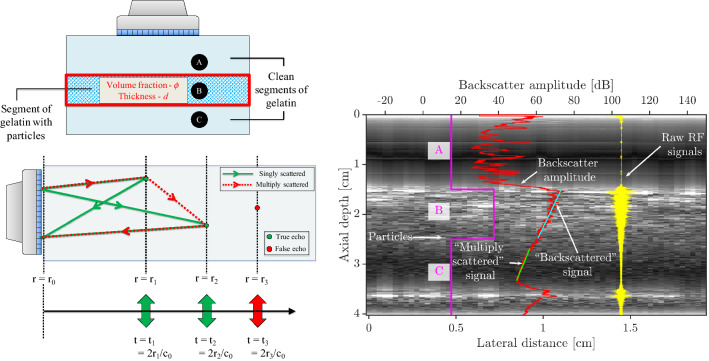
Evidence for the presence of multiple scattering. (Top left) Schematic of the gelatin based model. Particles are present only in segment B. (Bottom left) Simplified schematic of single and multiple scattering of sound propagating in a medium with a speed of sound $c_0$. (Right) Overlaid on the B-mode image is the profile of particle distribution. Shown also are the entire ensemble of raw RF signals as well as the backscatter amplitude. The backscatter amplitude signal can be further divided into the ‘backscattered’ signal (segment B) and ‘multiply scattered’ signal (segment C). The bottom wall of the gelatin model is located at an axial depth of about 3.6 cm

## Limitations induced by multiple scattering: tests in gelatin models

While empirical reconstruction techniques have been developed and applied, in previous studies (Furlan et al. 2012; Saint-Michel et al. 2017), the techniques were not validated with a known particle volume fraction profile. In principle, such a system can be created, wherein a box is divided into multiple partitions by means of vertical walls. However, preliminary experiments with such a system showed that the presence of the walls created additional issues, as they are not acoustically transparent. A possible solution would be to create walls with same acoustic impedances as the surrounding fluid (Ramotowski 2012), but this is not trivial and thus was not pursued. Gelatin based models were thus utilized, which are also commonly used in the field of medicine as low-cost phantoms to mimic tissue (Bude and Adler 1995; Culjat et al. 2010). In “Appendix D”, we provide further details on the practical intricacies involved in constructing these models. Moreover, only the stepwise reconstruction technique is considered for validation.

It must be noted that the nature of experiments in this section (particles immobilized in gelatin) is also central to the work of Fay et al. (1976). However, they investigate such models with the aim of recognizing inhomogeneities in the context of medical diagnostics. We also note that similar experiments with immobilized suspensions to quantify particle volume fractions have also been performed in the context of optical measurement techniques (Knowles and Kiger 2012; Liu and Kiger 2016) as well as magnetic resonance imaging (Borup et al. 2018).

### Multiple scattering in gelatin models

In the present work, we use gelatin as a medium to immobilize particles, allowing for construction of known particle volume fraction profiles. This allows us to employ ultrasound in a controlled environment where the particle volume fractions are known. A schematic of such a gelatin based model is shown in Fig. 8. The particle volume fraction profile in this schematic resembles a step function, composed of two clean segments of gelatin (segments A, C) with a particle-laden segment (segment B) sandwiched in between. Each segment may be characterized by its thickness, while the particle-laden segment may additionally be characterized by the volume fraction of the particles.

A major reason for venturing into the gelatin models was to have an environment without the presence of any walls that could contaminate the signal. These models also allowed for the detection of “multiple scattering”, depicted in Fig. 8. Multiple scattering is the phenomenon where a sound wave is scattered off multiple interfaces instead of a single interface. This leads to the sound wave covering a longer path and is received by the transducer at a later time. While the issue of multiple scattering is well known in various sub-domains of ultrasound/acoustic community (Tourin et al. 2000; Anugonda et al. 2001; Jia 2004; Snieder and Page 2007), this aspect has not been entirely addressed in the context of particle volume fraction profiling. The use of gelatin models provides easy access to isolate and visualize this phenomenon.

**Fig. 9:**
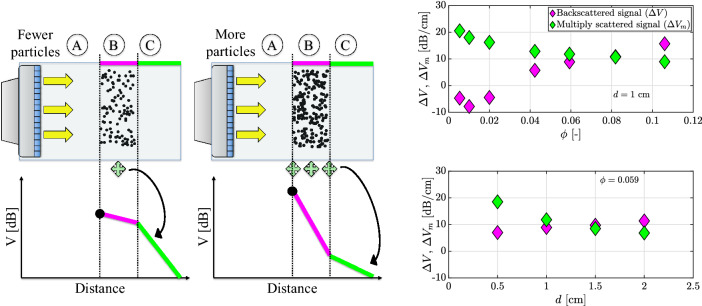
Characterization of the multiply scattered signal. (Left) Schematic for the behaviour of the backscatter amplitude profile as a function of the number of particles in a system corresponding to the schematic shown in Fig. 8. (Right) Characteristics of the backscatter amplitude profile ($\varDelta V, \varDelta V_m$) as a function of volume fraction ($\phi$) and thickness (*d*) of the particle-laden segment B

The right image in Fig. 8 provides strong evidence for the existence of this phenomenon in the gelatin system. The case considered resembles a step function, i.e. a particle-laden segment is sandwiched between two clean segments of gelatin. Shown in the background is a B-mode image. What is clearly visible in the B-mode image is the axial depth at which the particle-laden layer begins (about 1.5 cm) and the bottom of the gelatin model (about 3.6 cm). However, it is not very clear from the B-mode image where the particle-laden layer ends. Also shown is an ensemble of RF signals, as well as the backscatter amplitude profile, both of which provide clear evidence on the presence of multiple scattering. The multiply scattered tail is also referred to as ‘coda’ by seismologists (Snieder and Page 2007).

Three regions can be identified. The first region, segment A (axial depth between 0 and 1.5 cm), has no particles present which, per expectations, results in corresponding RF signals with low strength (due to a lack of scatterers) and a fluctuating backscatter amplitude profile. The second region, segment B (axial depth between 1.5 and 2.5 cm), has particles present in it. The RF signals too indicate the presence of scatterers in this region. Similarly, the backscatter amplitude profile can be characterized pretty well by means of a linear fit (to reiterate, actually exponential decay, but linear in decibel units), which is expected from a uniform suspension. The third region, segment C (axial depth between 2.5 and 3.6 cm), is again one without any particles. However, the RF signals indicate the presence of scatterers in this region and the backscatter amplitude profile also suggests that a uniform mixture may be present here, which is not the case. Thus, the observations from the third region confirm the existence of multiple scattering and it can be expected to cause issues in the volume fraction profile reconstruction if it is not accounted for. It must be noted that Fay et al. (1976) do not observe such a behaviour. A possible explanation for this is that they might have used lower volume fractions of particles in their experiments, which they have not reported explicitly.

### Characterizing the scattering behaviour

A disadvantage of gelatin models as compared to agitated suspensions is that the latter allows for a better ensemble averaging due to the inherent motion of the scatterers (Hall et al. 1997). It must be noted that only three measurements were made with 64 lines in each measurement. In order to obtain sufficient convergence in the backscatter amplitude data, each line from each measurement was utilized resulting in 192 ‘effective’ measurements. A shortcoming of these gelatin models is a lack of flow, which necessitates moving the transducer in order to probe more particles to reach statistical convergence.

Two straight line fits can be identified in the backscatter amplitude profile (Fig. 8) which are referred to as the “Backscattered” and the “Multiply scattered” signal, corresponding to segments B and C, respectively. A question that may be raised is how the characteristics of these fits behave as a function of the number of particles in the system. The number of particles can be modified by changing either the volume fraction of the particle-laden layer or its thickness. The results are illustrated in Fig. 9.

**Fig. 10:**
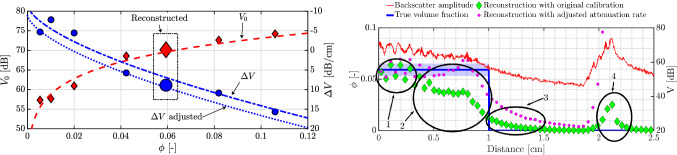
Reconstruction of a step particle volume fraction profile. (Left) Calibration curves for the gelatin models, based on segment B. The larger symbols represent the characteristics of the case on which we apply stepwise reconstruction. (Right) Application of the stepwise reconstruction process to the phantom (segments B and C). For the reconstruction with the above calibration, the following can be noticed: 1—Accurate reconstruction; 2—Error due to undercompensation; 3—Error due to multiple scattering; 4—Detection of container bottom

The slope of the backscattered signal as a function of the volume fraction is in line with the expectations (Fig. 3). The slope of the multiply scattered signal is seen to decrease with both the particle volume fraction as well as the layer thickness. This can be understood by means of the schematic in Fig. 9. The presence of more particles leads to the presence of more scattering events. The acoustic wave travels a tortuous path resulting in the transducer receiving signal for a longer period of time, i.e. appearance of particles beyond the particle layer via space-time correspondence. Thus, the rate at which the signal decays is slower when more particles are present. In fact, similar observations have been reported by Tourin et al. (2000).

However, the result that is most puzzling is that the slope of the backscattered signal consistently rises with the thickness of the layer, albeit weakly. That a trend exists is not surprising since multiple scattering would start affecting the signal beyond a certain thickness. This observation could also have implications on the accuracy of the stepwise reconstruction process. Calibration is usually performed over the entire size of the flow geometry, while the reconstruction is done on a much smaller region, which may lead to signal overcompensation while accounting for the attenuation losses. Javanaud and Thomas (1988) state that the critical depth beyond which single scattering theories become invalid in the intermediate wavelength regime can be approximated by $\sim a/\phi$. For a suspension with $\phi = 0.0593$ and $a = 265 \, \mu$m, the critical depth would be 0.45 cm. Thus, the effect of multiple scattering can be inhibited by imaging a small field-of-view, similar to Saint-Michel et al. (2017).

A caveat of these gelatin models is that attenuation due to absorption effects (see “Appendix B”) are expected to be much higher than in a liquid. However, there seems to be sufficient evidence that this does not afflict the present interpretation of data. For example, the backscatter amplitude profile in the the region 0–1.5 cm in Fig. 8 does not display a consistent attenuation behaviour. Moreover, the “multiply scattered” signal is not an artefact of this absorption since it is clearly dependent on the thickness and volume fraction of the particle-laden layer.

The above characterization implies that the presence of multiple scattering will complicate the accurate reconstruction of the volume fraction profiles. This would pose limitations on the critical depths and volume fractions that can be investigated while minimizing the influence of multiple scattering.

### An example of calibration and reconstruction

The first demonstration of the performance of the reconstruction technique is done by means of a step function profile for the particle volume fraction. Seven cases were available where the particle-laden segment had the same thickness (of 1 cm), while the particle volume fraction differed. Data corresponding to the particle-laden segment of 1 cm was then used for the generation of calibration curves (Fig. 10). Using the calibration data, an attempt was made to profile the particle volume fraction in one of the seven gelatin models ($\phi$ = 0.059), in the regions corresponding to segments B and C. Power-law fits were used for characterizing both the peak backscatter amplitude, as well as the amplitude attenuation rate, based on the signal corresponding to segment B.

The reconstruction attempt for a particle volume fraction profile having the shape of a step function is shown in Fig. 10. Shown also is the corresponding backscatter amplitude profile, which displays a clear periodicity in its shape. This is attributed to the method of preparation of these gelatin models where the particle-laden segment was created by individually immobilizing a 0.1 cm layer. Moreover, since only three frames were recorded, the periodicity is not suppressed. This periodicity is also transferred into the reconstructions. However, the periodicity seems to be diminished in the “multiply scattered” section of the signal. Two reconstruction curves are considered, both of which were extracted by the stepwise reconstruction approach.

The first reconstruction (green diamonds) is based on the calibration curves in Fig. 10. Four distinct regions can be identified for this reconstruction. In the first region, the reconstruction seems to work pretty accurately, apart from the periodic fluctuations (mean absolute error = 5.3 $\times 10^{-3}$, or 9% in a relative sense). This happens as the peak backscatter amplitude for this case (large red diamond in Fig. 10) nearly coincides with the curve fit (dashed red line). However, problems begin to arise in the compensation step of the stepwise reconstruction technique. As can be seen in the calibration curves, the amplitude attenuation rate for the phantom (large blue circle) is higher than predicted by the curve fit (for the volume fraction of the phantom, dash-dotted blue line). Due to this, the attenuation losses are undercompensated, which further leads to an underestimation of the volume fraction. Furthermore, this error propagates monotonically. This issue is highlighted in the second region. The error in the initial underestimation propagates slowly (0.4 - 0.8 cm), while it accelerates after a certain point (0.8–1.0 cm). This difference is caused by the different gradients in the calibration curve for the peak backscatter amplitude. The peak backscatter amplitude is more sensitive to volume fraction changes at lower volume fractions than for higher ones. The negative consequence of multiple scattering on the volume fraction profile reconstruction is visible in third region. Despite the absence of particles, a volume fraction profile is calculated, whose effect persists for approximately 0.4 cm. Finally, in the fourth region, an artefact caused by the bottom wall of the container becomes visible in the volume fraction profile.

The second volume fraction profile (magenta circles) is also built up using the stepwise reconstruction approach. However, in this case, the calibration curve for the attenuation rate has been artificially increased (by 2.5 dB/cm) so that the attenuation rate predicted by the curve fit nearly coincides with the attenuation rate of the phantom (dotted blue line). The reconstruction that follows resembles the step function much better. The mean absolute error for this reconstruction over regions 1 and 2 is $6.4 \times 10^{-3}$ (11% in a relative sense) as compared to $18.2 \times 10^{-3}$ (31% in a relative sense) for the previous one. This is due to the absence of errors caused by under/over-compensation for attenuation losses. However, in this case, the effect of multiple scattering lingers on much deeper into the model (> 1 cm).

This example already provides two major conclusions. Firstly, multiple scattering can contaminate the volume fraction profile reconstruction, and can blur gradients. Secondly, the goodness of the calibration curve fits is critical for the accuracy of the reconstruction. In these gelatin models, the quality of the calibrations were lower owing to the limited number of measurements, as well as the absence of moving particles. However, if calibration is performed in a suspension with moving particles (as will be done in the next section), the quality of the statistics is expected to improve. It is unmistakable that the quality of the backscattered signal in the gelatin model and the subsequent reconstruction is noisy. In our experience, despite careful, repeated attempts, such issues persist in the gelatin models, highlighting a shortcoming of the models themselves. Nevertheless, these models also help appreciate the limitations of the entire process in obtaining an accurate volume fraction profile.

## Application to particle-laden pipe flows

### Background to radial migration in particle-laden pipe flows

A very common application of ultrasonic particle volume fraction profiling is studying the flow of heavy particles (see “Appendix A”). This is typically accompanied with monotonically increasing particle concentration with depth. Here, we apply the technique to particle-laden pipe flows of a neutrally buoyant suspension, which is accompanied by a non-monotonic concentration profile. A practical application of this specific flow can be found in solid-liquid food flows (Lareo et al. 1997a, b).

This non-monotonicity is associated with the phenomenon of inertial migration/focusing, especially observed for a suspension with large particles in laminar flows. The reader interested in a detailed understanding of the phenomenon is directed to the seminal works of Segré and Silberberg (1962), Matas et al. (2004) as well as the recent works of Morita et al. (2017) and Nakayama et al. (2019). As summarized by Nakayama et al. (2019) for a dilute suspension: at low Reynolds number, particles are focused at the so called - Segré-Silberberg annulus, approximately 0.3 pipe diameters away from the pipe axis. Upon increasing the Reynolds number, this annulus moves towards the wall, while another annulus concentrated with particles begins to appear closer to the pipe axis, the so called inner annulus. At even higher Reynolds numbers, the Segré-Silberberg annulus ceases to exist, and particles aggregate only at the inner annulus. Moreover, this phenomenon requires an extremely long development length (over a thousand pipe diameters).

The above is valid for dilute suspensions ($\phi \sim 10^{-4}$) with non-existent particle-particle interactions, where the suspensions were studied by standard optical means. The migration of particles away from the axis is attributed to fluid-particle interactions. Endeavours at studying this phenomena at higher volume fractions ($0.06 \le \phi \le 0.45$), albeit at much lower Reynolds numbers have also been made, with techniques such as refractive index matching (Koh et al. 1994), electrical impedance tomography (Butler and Bonnecaze 1999), as well as magnetic resonance imaging (Hampton et al. 1997; Han et al. 1999). A common qualitative observation, at least for $\phi \ge 0.20$, was the migration of particles towards the pipe axis, which is attributed to particle-particle interactions. As a consequence of this migration towards the pipe axis, the velocity profile changes from a parabolic shape to a blunted one.

### Experiments

We apply the stepwise reconstruction technique to particle-laden pipe flows for a wide span of Reynolds numbers and bulk volume fractions (laminar, intermittent as well as turbulent regimes for $$0 \le \phi _{\rm{bulk}} \le 0.09$$). All experiments are performed in a plexiglass pipe with an internal diameter of $$D = $$ 10.00 mm with a neutrally buoyant suspension composed of Polystyrene particles (diameters, $$d_p = $$ 530 ± 75 $$\mu $$m) in a salt-water solution, resulting in $$D/d_p \approx 18.9$$. The flow is gravity-driven with the flow entering the tube from a reservoir via an inlet chamber and measurements are performed 270*D* downstream of the inlet. A slightly adapted version of this facility was also utilized in the study of Hogendoorn and Poelma (2018).

A different ultrasonic apparatus is used in the experiments shown below, namely a Verasonics Vantage 128 in combination with a linear probe (L11-5v). This transducer too has 128 individual piezoelectric elements spanning $\sim$ 3.9 cm. The ultrasound transducer is immersed in a water bath surrounding the pipe, to aid acoustic coupling. Imaging is performed with an ultrasonic central frequency of 10.5 MHz. Data are sampled at 62.5 MHz with a 14-bit resolution. This apparatus provides an alternative to the conventional ultrasound imaging techniques: plane-wave imaging. This change is warranted by the fact that turbulent flows are extremely dynamic processes involving small time-scales. In order to study such flows, the plane-wave imaging technique offers the possibility to acquire images in the kilohertz range and accurately quantify the turbulent characteristics of the flow (Hogendoorn and Poelma 2019). The post-beamformed RF data were further converted to B-mode images, which were then utilized for the volume fraction profiling. B-mode images are more readily accessible and are commonly used for velocimetry analyses. For each experiment, at least four separate measurements were made consisting of 10000-20000 images recorded at 400-4000 frames per second. For the calibration cases, all recordings were at 4000 frames per second. In order to assess the repeatability, the flow was stopped and restarted between each of the four ensembles.

**Fig. 11:**
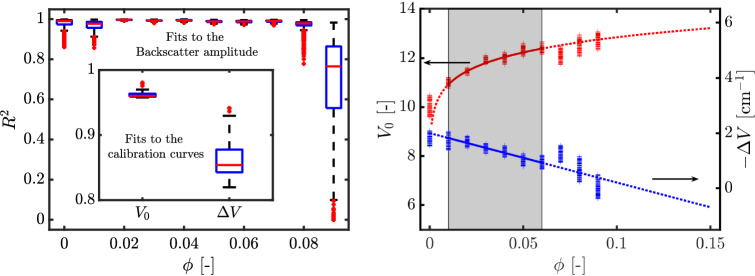
Characteristics of the empirical fits for particle-laden pipe flow. (Left) Coefficient of determination for linear fits to the backscatter amplitude profile. The values in the inset are the coefficients of determination for the fits to the peak backscatter amplitude and the amplitude attenuation rate. (Right) Calibration curves—power law for the the peak backscatter amplitude and first-order polynomial for the amplitude attenuation rate. Individual markers represent the individual 128 piezoelectric elements and the lines are based on the mean value

**Fig. 12:**
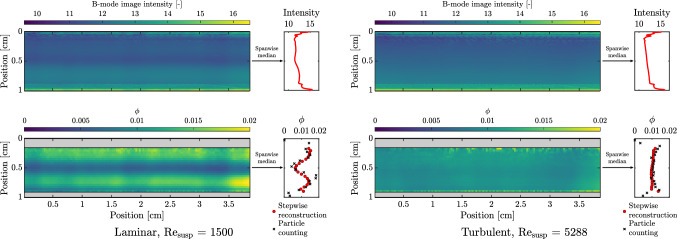
Comparison of time-averaged B-mode intensity images and reconstructed particle volume fraction profiles from the calibration. Two cases are considered, both for $\phi _{\rm{bulk}} = 0.01$—a laminar (Re$_{\rm{susp}}$ = 1500) and a turbulent one (Re$_{\rm{susp}}$ = 5288)

### Calibration in uniform suspensions

Before applying the technique to non-uniform suspensions, calibration parameters (peak backscatter amplitude and amplitude attenuation rates as a function of bulk volume fraction) were estimated for each of the 128 transducer elements, as shown in Fig. 11. The scatter in the estimated coefficients suggests that using a calibration for each individual element instead of a global statistic such as a mean/median would improve the accuracy of the reconstruction. For the present discussion, calibration fits are performed only for for $0.01 \le \phi \le 0.06$ by fitting a power law for the peak backscatter amplitude and a first order polynomial to the amplitude attenuation rate. These curves are extrapolated to the range of $0 \le \phi \le 0.15$. The coefficients of determination for the linear fits to the backscatter amplitude profiles are mostly acceptable, however, this does not hold true for the calibration curves, especially for the amplitude attenuation rate. As a consequence, it can be expected that errors due to an imperfect compensation for the attenuation could accumulate/propagate and result in erroneous quantification of the volume fractions. Another observation is that the attenuation is negative and this arises due to the usage of time gain compensation (see Bushberg and Boone 2011, Fig. 14–27, for an illustrative example) which electronically increases the backscatter amplitude in depth.

### Reconstruction in dilute suspensions: comparison with particle counting

First, we consider the reconstruction of the volume fraction profile in a dilute suspension ($\phi _{\rm{bulk}} \sim 0.01$). Under such conditions, individual particles are still discernible in the image. In order to validate the stepwise reconstruction technique, a comparison of the reconstructed volume fraction was done against that from the particle counting technique. Particle counts were then converted to a volume fraction in an ad-hoc manner by assuming the bulk volume fraction of the turbulent case to be 0.01, verified as a reasonable choice by collecting a sample of the suspension.

Two representative cases are considered in Fig. 12: one under laminar flow conditions and the other under turbulent conditions. By considering the B-mode images, a qualitative difference between the two is visible in the form of an abnormal intensity gradient under laminar conditions in the vicinity of the pipe axis ($\sim$ 0.5 cm), which becomes more evident in the spanwise median intensity profile. Such an abnormality is absent under turbulent conditions and the spanwise median intensity profile is characterized by a straight line, which is an indication of the uniformity of the dispersion that is exploited for calibration.

These differences in the image intensities translate into differences in the volume fraction reconstructions. It is vividly clear that the phenomenon of inertial migration is present under laminar conditions. Very similar profiles have been recorded in ultrasonic backscattering measurements of laminar blood flow, and has been called “black hole” (Yuan and Shung 1989; Qin et al. 1998). The comparison of the reconstruction to the particle counting technique helps in the verification of this phenomenon and that it is not an artefact of the reconstruction. The mean absolute difference between the estimates by the two techniques, for these two examples, is approximately $1.5 \times 10^{-3}$ (or 15% relative difference, with respect to the mean bulk volume fraction), confirming good performance by the stepwise reconstruction. In comparison, the turbulent flow has a relatively uniform distribution of particles. Thus, qualitative information about particle volume fraction profiles may also be extracted from the local echo intensity/amplitude (for example, see cluster IV in “Appendix A”). However, for quantification purposes, the attenuation would have to be accounted for appropriately.

**Fig. 13:**
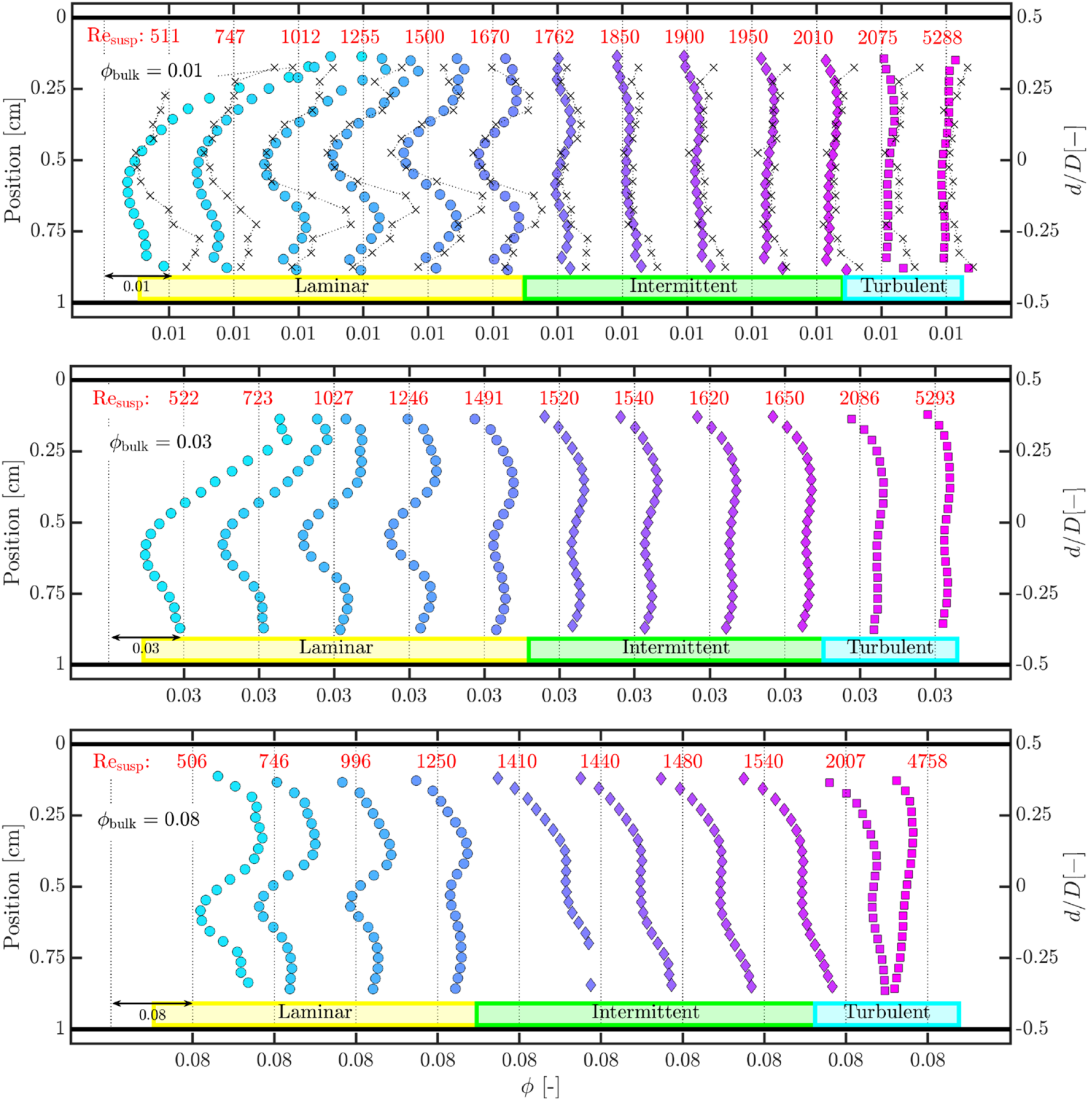
Compilation of reconstructed time-averaged particle volume fraction profiles for flows with bulk particle volume fractions of 0.01, 0.03 and 0.08. Missing markers indicate that estimated volume fraction is outside the range $0 \le \phi \le 0.15$. For $\phi _{\rm{bulk}} = 0.01$, results based on particle counting are also shown (dotted line with crosses)

### Application of technique to higher volume fractions

Finally, we apply the stepwise reconstruction technique to higher volume fractions, as well as to several Reynolds numbers. A detailed compilation of constructed volume fraction profiles for three bulk volume fractions is shown in Fig. 13. The Reynolds numbers considered the entire span of flow regimes: laminar, intermittent and turbulent. These reconstructions are performed on the median intensity along the spanwise direction of the B-mode image, with help of the mean of the fitting coefficients for the 128 individual line elements. Only volume fractions in $0.01 \le \phi \le 0.06$ are used for characterizing the uniform suspensions, implying that all reconstructions with $\phi > 0.06$ are extrapolations (dashed lines in the right image of Fig. 11). In the current context, all the Reynolds numbers reported here are based on the bulk velocity ($U_{b}$), pipe diameter (*D*), kinematic viscosity of the suspending fluid ($\nu _{f}$) and relative viscosity ($\chi ^{e} = (1+1.25\phi /(1-\phi /0.64))^{2}$) incorporated in them. The relative viscosity quantifies the effective viscosity of the suspension with respect to the suspending fluid and is based on a formulation first proposed by Eilers (1941). The appropriateness of this fit for the current suspension has been shown by Hogendoorn and Poelma (2018), and the Reynolds number is defined as $\text{Re}_{\text{susp}} = {U_{b}D/(\nu _{f} \chi ^{e})}$.

Three bulk volume fractions are considered ($\phi _{\rm{bulk}}$ = 0.01, 0.03, 0.08), all of which may be considered in the dilute/semi-dilute regime. In the semi-dilute regime, particles are also able to interact with each other by means of hydrodynamic forces (Guazzelli and Pouliquen 2018). Reconstruction of the volume fraction profiles for all the three cases can be characterized with a common observation. Laminar flows typically show a deficit in the volume fraction near the pipe axis with local peaking off-axis. This behaviour of reduced particle volume fractions near the pipe axis persists even for flows with low levels of intermittency (defined as fraction of flow with puffs). With increasing levels of intermittency and eventually entering the turbulent flow regime, the non-uniformity gradually diminishes, especially for $\phi _{\rm{bulk}} = 0.01$. While the profile for the highest Reynolds number for $\phi _{\rm{bulk}} \sim 0.01$ appears rather uniform, there still appears to be a weak non-uniformity for $\phi _{\rm{bulk}} \sim 0.03$.

The most noteworthy observation common to all three cases is the gradual disappearance of the distinct core with reduced volume fractions, upon increasing the Reynolds number. Prior to interpreting the physical significance of this observation, it is worth considering whether or how it could be affected by the various sources of error. Fortunately, the flow inherently allows us to sample more unique instances in comparison to a static gelatin model. Among the sources of error identified earlier in the manuscript, the chief are: untrustworthy signal in the near-wall region, imperfect calibrations as well as multiple scattering. Errors caused by imperfect calibrations could exacerbate any errors generated by the untrustworthy signal in the near-wall region.

We start with multiple scattering. This phenomenon plays a stronger role with increasing volume fractions, and can primarily blur gradients. Thus, for $\phi _{\rm{bulk}} = 0.08$, the gradients may appear smoother than in reality, while this phenomenon is expected to only minimally affect the reconstructions for $\phi _{\rm{bulk}} = 0.01, 0.03$. The independent effect of an imperfect calibration is best visible in the reconstruction for $\phi _{\rm{bulk}} = 0.08$ and $Re_{\rm{susp}} = 4758$. This is the data used for calibration. The deviation of the actual measurement points from the extrapolated calibration curve (right subfigure in Fig. 11) induces an error in the reconstruction. The attenuation is overcompensated for, which leads to a gradually decreasing estimation of the particle volume fraction. For $\phi _{\rm{bulk}} \sim 0.08$, $1410 \le$ Re_susp_ $\le 2007$, the volume fraction profiles are accompanied with a linear trend. These could arise from an initial error in the estimation of the volume fraction, generated by the untrustworthy signal, in tandem with an inappropriate compensation of the attenuation, with the error compounding with depth. Notwithstanding this linear trend, a local minimum in the volume fraction profile appears, albeit a weak one.

As a final step to convince that the ultrasonic reconstructions are qualitatively sound, particle volume fraction profiles estimated by particle counting are also presented for $\phi _{\rm{bulk}} = 0.01$. There is a good agreement between the two reconstructed profiles suggests that while there are quantitative discrepancies, reconstructions using ultrasound are qualitatively accurate. As was emphasized in Sect. 5.4, there is a direct correlation between the reconstructed volume fraction profile and the corresponding B-mode image intensities. Of course, for the lower Reynolds numbers, there is a sharp rise in the local volume fraction, which could also deteriorate the accuracy of the particle counting technique. In short, we feel that despite the various sources of error which quantitatively affect the reconstruction to varying degrees, the gradual disappearance of the distinct core with reduced volume fractions upon increasing the Reynolds number is physical.

This behaviour of the distinct core with reduced volume fractions is likely the signature of radial inertial migration of particles that have been observed in extremely dilute suspensions, under laminar conditions. Han et al. (1999) have observed similar concentration profiles in their magnetic resonance imaging measurements for $\phi = 0.06, 0.10$, albeit at very low Reynolds numbers (see Figs. 7a, 9a therein). Maude and Yearn (1967) too report a similar behaviour (see Fig. 10 therein) for comparable experimental parameters ($D/d_p \approx 21.7$, Re_susp_ $\approx 480$, $\phi \approx 0.07$), but at a much lower streamwise location. In this context, it is also worthwhile to mention that such a depleted core has also been observed in numerical simulations of particle-laden laminar, channel flows for $\phi \le 0.1$ (Kazerooni et al. 2017). A key difference, however, is that the numerical simulations suggest a complete depletion unlike the present reconstructions. Of course, it must be noted that the reconstructed volume fraction profiles considered here are at a fixed distance of 270*D* downstream of the inlet. It would be worthwhile to make measurements at several locations in a longer pipe, in order to conclude whether the velocity *and* particle concentration profiles are fully developed or not.

In contrast, there are far fewer studies that explicitly report particle volume fraction profiles in the transitional or turbulent flow regime for comparable experimental parameters. Our reconstructions suggest that the distinct core even persists into the transitional flow regime. Studying these regimes with multiple measurement techniques as well as fully-resolved simulations will be required for a complete understanding of the flow.

## Conclusions and outlook

The aim of this manuscript was to address the possibility to extract information on particle volume fraction profiles in dispersed multiphase flows by means of ultrasonic waves. We focused specifically on empirical approaches wherein the reliance upon theoretical models describing the interaction between ultrasound and suspensions is circumvented. Such techniques can be advantageous for studies where an available theoretical model may not be readily applicable (for example, when using linear array transducers or while investigating flow geometries with walls).

We discuss two possible ways to extract quantitative particle volume fraction profiles: stepwise reconstruction (Sect. 3.1) and dual-frequency inversion (Sect. 3.2). Both these techniques are reliant on a calibration procedure, which is performed in uniform suspensions (Sect. 2), yielding two key volume fraction dependent parameters: the peak backscatter amplitude and the amplitude attenuation rates (Figs. 3 and 4). We explore the applicability of these empirical methods by, first, testing the inversion techniques on synthetically generated data (Sect. 3.3) and identify that errors generated in the near-wall regions are likely to inhibit the quantitative performance of the reconstructions, especially for the stepwise reconstruction technique.

Because of the detrimental effect of the walls, we next applied the stepwise reconstruction technique to a particle-laden gelatin model (Sect. 4). This helped appreciate the need for a prudent choice for calibration curves as well as isolate the presence of multiple scattering (Figs. 8 and 10). The phenomenon of multiple scattering limits the imaging depth as well as particle loading, while also reducing the accuracy of the technique, especially for concentration gradients. Our introduction of gelatin models in the context of particle volume fraction profiling using ultrasound can be useful. However, despite careful attempts, it proved challenging to obtain good quality data.

Lastly, we apply the stepwise reconstruction technique to particle-laden pipe flows with bulk volume fraction as high as 0.08 (Sect. 5, Fig. 13). We unveil the presence of a core with a deficit of particles, most likely associated with the phenomenon of inertial migration. This core becomes less pronounced with increasing Reynolds number, yet persisting into the intermittent flow regimes, while there is also weak evidence of it sustaining into the turbulent flow regime.

The techniques presented here also have potential for being improved further. Dedicated acoustic simulation software exist (primarily from the medical field) that can be used to generate ultrasonic images. Examples include Field II (Jensen 1997) and k-Wave (Treeby and Cox 2010). While the former is restricted to point scatterers, the latter can also accommodate volume occupying spheres with custom properties such as mass density and speed of sound. The latter could thus serve as a possible tool for generating realistic synthetic images relevant for particle-laden flows, which could prove useful in assessing the accuracy of the particle volume fraction reconstruction techniques. Another issue that needs to be tackled is a practical manner to overcome issues arising from multiple scattering. In fact, advances along this line have already been made for static media (Aubry and Derode 2009). And, while the profiling techniques presented here may be extended also to emulsions, bubbly flows would warrant alternative approaches (Murai et al. 2009). Finally, the shape of the ultrasound beam can also be assimilated into the analysis to improve the estimated volume fraction profiles.

In closing, this manuscript highlights possible empirical approaches for quantifying/assessing particle volume fraction profiles in particle-laden flows, while also elaborating upon limiting factors that compromise the quantitative accuracy. In any case, ultrasound particle volume fraction profiling, especially in tandem with ultrasound based velocimetry, can be instrumental in unravelling the local characteristics of particle-laden flows.

## Appendix A: Existing acoustic/ultrasonic concentration profiling techniques

**Table 3:** Overview of studies targeting ultrasonic particle volume fraction profiling in particle-laden flows. Six clusters have been made primarily on basis of the features of the technique

Work(s)	Technique	Experiments
(I) *Techniques developed originally for estimation of suspended sediment concentrations in marine environments*		
Thorne and Hanes (2002), Hurther et al. (2011), Thorne et al. (2011) and associated works	• Technique developed and refined over a span of decades	• Technique is typically used for mass concentrations of 1-10 kg/m^3^ which corresponds to $\phi \sim 0.005$
	• Meticulous inclusion of acoustical scattering theory into methodology	• Technique constrained to fixed combination of particles and ultrasound transducer
(II) *Techniques for Acoustic Backscatter Systems applicable to arbitrary suspension flows*		
Admiraal and García (2000), Hunter et al. (2012b), Rice et al. (2014), Bux et al. (2015), Rice et al. (2015) and associated works	• Semi-empirical approach (needs calibration)	• Primarily applied to settling suspensions and pipe flows of heavy particles suspended in a liquid medium
	• Theoretical formulations adapted from cluster I extended to arbitrary suspensions	• Domain sizes ranging from $\sim$ 4 to $\sim$ 40 cm
		• Technique typically applied for $\phi _{\rm{bulk}} < 0.03$
(III) *Purely empirical techniques (calibration in uniform suspensions then exploited for non-uniform suspensions)*		
Furlan et al. (2012), Saint-Michel et al. (2017), *present work*	• Calibration parameters obtained in uniform suspensions	• Experiments in pipe (2.5 cm) and Taylor-Couette geometry (0.2 cm)
	• Non-uniform suspensions studied with help of calibration	• Uniform suspensions, $\phi _{\rm{bulk}} \le 0.15, 0.40$
	• Account for attenuation	• Non-uniform suspensions, $\phi _{\rm{bulk}} \le 0.10, 0.15$
(IV) *Direct correspondence between echo intensity and particle volume fraction (empirical, but not accounting for attenuation)*		
Zou et al. (2014), Gurung and Poelma (2016), Stener et al. (2014), Stener et al. (2016), Hitomi et al. (2018), Saint-Michel et al. (2018)	• Directly correlate (power spectral density of) backscattered echo intensity to local volume fraction	• Domain sizes varying from $\sim$ 1 to $\sim$ 40 cm
	• Do not account for attenuation	• Maximum volume fractions studied between $0.01 \le \phi _{\rm{bulk}} \le 0.10$
	• Calibration can be done in uniform suspensions	
(V) *Particle counting*		
Chemloul et al. (2009), Lee et al. (2018)	• Identify individual particles	• Typically applicable to dilute suspensions $\phi _{\rm{bulk}} < 0.02$
	• No calibration needed	
(VI) *Pair(s) of separate transmitter-receiver configuration (typically use relations for time-of-flight and attenuation)*		
Gunning et al. (1988), Wedlock et al. (1993), Hoyos et al. (1994), Pinfield et al. (1996) and other works	• Traverse pair perpendicular to direction of wave propagation (non-intrusive)	• Usually applied to extremely slowly sedimenting/creaming flows (over days)
	• Alternatively, install several pairs	• Volume fractions as high as $\phi _{\rm{bulk}} \sim 0.6$ studied
Warsito et al. (1997), Vatanakul et al. (2004)	• Traverse pair through the flow in direction of wave propagation (intrusive)	• Often in context of three-phase flows • Domain sizes $\sim$ 10 cm
Warsito et al. (1999), Utomo et al. (2001)	• Several pairs used (non-intrusive)	• In context of three-phase flows
	• Computed Tomography algorithms used for reconstructing 2-D profiles	• Domain sizes $\sim$ 10 cm

The topic of volume fraction profiling using ultrasound has already been the subject of several studies as evidenced by the summary in Table 3. In this table, the works have been categorized into six clusters (I-VI), primarily based on the nature of the technique. It is possible that we may have missed a few studies in this summary owing to the wide variety of fields where ultrasound finds application. Nevertheless, we hope that this table can offer the readers a relevant reference depending on their application, field-of-view and apparatus at their disposal.

Works in cluster I can be considered to be very comprehensively developed methods with acoustical scattering theory at its heart. These have been specifically developed in the context of studying suspended sediment concentration profiles in marine environments, typically with the aid of bespoke Acoustic Backscatter Systems and/or Acoustic Concentration and Velocity Profilers. It must be noted that this topic is an independent field of research in itself, and there are several other papers that we have not included here.

A simplified version of the key equations central to the works in cluster I is shown in Eq. 5.

5 $$\begin{aligned} V(r) \propto M^{1/2} \exp \left (2\int _0^r \xi (r^{\prime })M(r^{\prime })dr^{\prime } \right ) \end{aligned}$$

This concisely elucidates the interaction between the measured, backscattered ultrasound signal, *V*(*r*), and the desired quantity, the distribution of the suspended sediment, *M*(*r*). Here, *r* is the depth, and $\xi$ is the attenuation coefficient. In short, *V*(*r*) is the rms of the voltage received, corresponding to the backscattered acoustic signal at a given depth *r*. This backscattered signal is proportional to the local mass fraction ($\propto M^{1/2}$) and also the net attenuation of sound until that depth (expressed by the integral). The exponential term indicates the exponential decay of the acoustic waves. It must be noted that the integral term also includes the local mass fractions, which complicates the equation.

At least three techniques to extract mass fraction profiles have been used (Hurther et al. 2011) - the iterative implicit inversion method, the explicit inversion method and the dual-frequency inversion method. In fact, most of the techniques developed in clusters II, III and even in this work may be considered to be generalized variants of the methods developed in cluster I. However, exact relations developed for cluster I are limited to marine sediment being sampled by an Acoustic Backscatter System with an eye towards application in field experiments. Moreover, the theory has been reported to be inapplicable beyond loadings of 2.5 g/L or $\phi \sim 0.001$ (Hunter et al. 2012a), due to multiple scattering (scattering of sound waves between particles).

Thus, the group of works in cluster II overcome a few constraints of this theory and extend the above formulations as well as inversion techniques to arbitrary suspensions (i.e. suspensions other than sediments in marine environments), while using Eq. 5 as basis. Their primary applications have been geared towards sediment flows/transport and in a recent endeavour, an array of acoustic backscatter systems was deployed in profiling a large-scale laboratory experiment (Hunter et al. 2020). However, as argued by Saint-Michel et al. (2017), the validity of Eq. 5 might breakdown at higher volume fractions, due to multiple scattering, which means that the techniques in cluster II may be considered semi-empirical. Moreover, the expression cannot be directly applied to linear array transducers as the manner in which the beam spreads would be different, and could also be a function of various imaging settings. In this spirit, the works in cluster III can be considered to be the most generalized and completely empirical methods which lump the acoustical scattering behaviours into a couple of calibration parameters (obtained in uniform suspensions). These may be applied to arbitrary suspensions with arbitrary beamforms. Moreover, such an approach can accommodate wall-bounded flows, as walls can be difficult to account for in the approach based on cluster I (Admiraal and García 2000). The techniques discussed in this manuscript can best belong to cluster III, with the common theme being the execution of calibration in uniform suspensions, with these being exploited in studying non-uniform suspensions. It must be noted that this step of calibration is also necessary in procedures involving cluster II.

Other methods based on echo intensity, cluster IV, and particle detection, cluster V, have also been proposed. While the latter technique is best used for dilute suspensions, the works based on echo intensities often do not compensate for attenuation of the sound in the reconstruction. Finally, works in cluster VI employ a separate transmitter and receiver. If only one pair is used, profiles can be determined by traversing the pair perpendicular to the direction of sound propagation at a fixed separation (non-intrusively) or parallel to it (intrusively). However, by using multiple pairs, Computed Tomography algorithms can be employed for reconstructing an entire cross-section. These studies are often performed in the context of gas-liquid flows (Hoyle 1996), with fewer examples involving solid particles.

## Appendix B: Interactions between ultrasound and suspensions

While ultrasound finds its most noteworthy applications in the field of medicine, it has also found applications in industrial settings. For example, ultrasound is utilized for systems involving (colloidal) suspensions and emulsions (Dukhin and Goetz 2002). One specific example is in the food industry where ultrasound finds widespread application in monitoring as well as processing of food products (Awad et al. 2012). It is thus unsurprising that very elaborate and rigorous theoretical models exist which describe the interaction between ultrasound and suspensions. The most well-known ones are ECAH (named after the authors’ initials Epstein and Carhart 1953; Allegra and Hawley 1972) and coupled-phase models (Harker and Temple 1988; Gibson and Toksöz 1989; Challis et al. 2005). These theoretical models are commonly used in commercial products to estimate particle size distributions (Challis et al. 2005). Below, we briefly discuss the most relevant aspects concerning the interaction between ultrasound and suspensions.

At a single-particle level, the interaction between the particle and a plane ultrasonic wave is governed by the relative wavelength *ka*, the product of the wavenumber ($k = 2\pi /\lambda$, where $\lambda$ is the ultrasonic wavelength) and scatterer radius (*a*). Mathematical investigations have been performed for cases where the scatterers are much smaller than the wavelength (Rayleigh 1896) as well as where they are of comparable sizes (Faran 1951). For example, in the ‘long wavelength regime’ ($ka \ll 1$), the scattering is dependent on the ultrasonic frequency, while this does not hold true in the ‘short wavelength regime’ ($ka \gg 1$). The work presented in this manuscript is primarily in the so called ‘intermediate wavelength regime’ ($ka \sim 1$), since we utilize a non-colloidal dispersed phase ($a \sim 0.2$ mm). In this intermediate wavelength regime, it is known that single spherical particles return rather complicated ultrasonic images, with a single particle returning echoes longer than the particle size itself (see Baddour et al. 2005, Fig. 4d, for an illustrative example).

A suspension is composed of a liquid continuous phase and a dispersed phase (rigid spherical particles in the present study). The continuous phase is usually homogeneous and primarily affects the acoustic waves by absorption (an irreversible process by which acoustic energy is dissipated into heat) whereas the dispersed phase can also affect the acoustic waves by other phenomena such as scattering (redirection of energy instead of dissipation). Both these mechanisms collectively lead to the extinction of the acoustic wave.

The absorption mechanism is typically associated with relaxation processes, where the medium returns to its original state after being exposed to a pressure variation induced by the sound wave (Bjørnø 2017). On the other hand, several mechanisms can be identified for the interaction between sound waves and colloids (Dukhin and Goetz 2002). For large particles ($ka > 1$), absorption mechanisms are negligible and the extinction of the acoustic waves may be attributed primarily to scattering processes. If the present study were to be concerned with colloidal particles (resulting in the long wavelength regime), the scenario would be the opposite, with negligible scattering and domination of absorption mechanisms. Thus, the profiling techniques discussed here may not be trivially extrapolated to colloidal suspensions.

## Appendix C: Basics of ultrasound imaging

Below, we briefly summarize the principles behind ultrasound imaging. The reader interested in a more extensive understanding of these principles and terminologies is referred to the review article by Poelma (2017) or the book by Szabo (2004), which includes the mathematics behind the imaging.

Ultrasound imaging is based on the use of acoustic waves (longitudinal waves) as compared to electromagnetic waves (transversal waves) used in optical techniques. At the core of the instrumentation responsible for the generation/reception of ultrasonic waves ($> 20$ kHz, beyond the range of human hearing) lie piezoelectric elements (capable of converting electrical signals into pressure waves and vice versa).

The general principle behind ultrasound imaging can be summarized with the phrase “pulse-echo”: the piezoelectric element is excited by electronic signals to generate pressure ‘pulses’ (characterized by central frequency and number of cycles) which traverse through the medium of interest as a beam. In a linear array transducer, several individual piezoelectric elements can be simultaneously employed to customize beam profiles (for example, to focus the beam at a given depth or steer it in a desired direction). Presence of scatterers/heterogeneities in an otherwise uniform medium, whose acoustic properties deviate from the surrounding medium, are responsible for generating ‘echoes’ (reflection of acoustic waves towards the ultrasound transducer). These echoes are then received by the piezoelectric element and its mechanical vibrations are converted to electronic signals. Since the same transducer is used for transmitting and receiving the ultrasonic waves, it is also referred to as a transceiver.

The initial electronic waves received are typically referred to as pre-beamformed RF (Radio Frequency) data. These electronic signals then undergo a receive beamforming process to return post-beamformed RF data. Receive beamforming refers to the process of applying delays to the echoes received by an array of individual piezoelectric elements to account for differences in times-of-flight such that their sum originates from a focal point (see Szasz 2016, Fig. 2.3(b), for an illustrative example). This step improves the image quality and resolution.

The post-beamformed RF data are the most basic form of ultrasound data that can be used further. This RF data are an alternating sinusoid (as the transmitted pulse is sinusoid) whose amplitude and phase is changing. Information about the scatterers/reflectors is contained in the amplitude of the RF data. Thus, an amplitude demodulation step (which includes envelope detection) is performed to retain the amplitude while getting rid of the sinusoidal oscillations. This new signal is referred to as A-mode (‘A’ for amplitude). The A-mode data is seldom used in the medical field and is commonly log-compressed to obtain B-mode data (‘B’ for brightness), resulting in greyscale images, which can be used by physicians for diagnosis. The log-compression step reduces the dynamic range resulting in a better balanced histogram of image intensities.

The B-mode data are a concatenation of multiple 1-D signals to form a 2-D array/image. These 1-D signals are time series based on a sampling frequency. The sampling frequency differs from the aforementioned central frequency, in that the latter describes the properties of the transmitted pulse, while the former characterizes the rate at which the echoes are electronically sampled. These 1-D time series can be converted to depths by utilizing the speed of sound of the medium.

The two-dimensional array/image has two directions: the direction parallel to the array of transducer elements is referred to as the lateral direction and the one perpendicular as the axial direction. While ideally, it would be desirable to have a thin, rectangular field-of-view (like in Particle Image Velocimetry), the actual region insonified by the transducer has a much more complex geometry (see Poelma 2017, Fig. 2, for an illustrative example). Importantly, the beam profile also has a thickness in the third dimension, also known as the elevational direction, resulting in the beam profile having a certain thickness.

Another aspect is that the beam profile has two distinct regions: a near-field (where the beam converges) and a far-field (where the beam diverges). The pressure amplitude characteristics in the near-field are non-monotonic and complicated, in comparison to the far-field where the ultrasound intensity of the field decreases monotonically with distance. Thus, it is typically desirable to image in the far-field of the ultrasound transducer.

A sequence of images can be recorded, with each individual image referred to as a frame. The temporal separation between the frames determines the frame rate.

## Appendix D: Recipe for the gelatin models

As mentioned in Sect. 4, the use of gelatin as a tissue mimicking substance is a common choice. Thus, there are several works which have their own “recipe” for creating a gelatin based phantom. Below, we describe our approach and experiences concerning the procedure for creating the gelatin models.

Commonly, we start with a certain quantity of a solvent (water or aqueous glycerol) and adding food-grade gelatin powder (Dr. Oetker GmbH) in a ratio of 20 grams per litre of solvent. Supposedly, gelatin is insoluble in glycerine, and if aqueous glycerol is used as a solvent, the resulting mixture may be characterized as “a very fine gelatin sponge containing glycerine in its pores” (Ridout 1879). The system is allowed to stay at rest for several minutes to allow the gelatin particles to swell. Hereafter, the system is stirred with the aid of a magnetic stirrer, while simultaneously warming it from underneath (temperatures typically between 50 and 90 °C). The process of warming and stirring is continued until the gelatin particles dissolve and a clear, transparent solution is obtained. A few drops of Tween-20 are also added to aid the wetting of the particles that shall be introduced later. It is recommended to stir the solution at moderate rates, otherwise at higher rates air bubbles could get entrained into the solution, which can be difficult to get rid of, if the solution is highly viscous. On one occasion 50 grams of gelatin per litre of solvent was tested, which resulted in a longer waiting period for the dissolution of gelatin as well as the solution retaining a yellowish tinge, both of which are undesirable. However, this would increase the stiffness of the resulting model.

Once the solution is prepared, the desired particle-laden model can be prepared. Typically, we build up the model in thin sheets. Basically, only a very small volume of solvent is mixed with the appropriate amount of particles (based on desired volume fraction) and is added to the container corresponding to a sheet with a thickness of about 1–1.5 mm. An exception to this is the bottom clean segment, or segment C, in Fig. 8 which is created in one iteration. The boundaries between the sheets are sometimes clearly visible in the ultrasound images, implying marginal acoustic impedance variations across the sheets. A few drops of food color can also be introduced to create an easy visual reference of the model (Fig. 1). Hereafter, this sheet of solution is allowed to gel by placing the container in a refrigerator ($\sim 5 ^{\circ }$C). The sol-gel transition temperature has been found to be in the range of 20–30 °C (Parker and Povey 2012). One thing that could occur, while adding the solution, especially with a syringe or pouring from a height, is the entrapment of air bubbles. Thus, it is recommended to introduce the new batch of solution as gently as possible. In case it is difficult to remove any bubbles, they are pushed to one of the walls and eliminated with the help of a spatula. The temperature of the to-be-added solvent should be optimal (60–70 °C in our experience). If it is too low, it is highly viscous and does not spread readily over the gel surface and gels quickly, resulting in an uneven surface. On the other hand, if the new solution is too hot, it could erode the gel surface underneath. Typically, the transition of the newly introduced solution to gel would take only a few minutes for thin sheets. If insufficient time is allowed for this solidification process, particles can move across sheets of solutions, in case there are density differences between the particles and the gelatin solution.

Often, we prepared several containers, with varying profiles, simultaneously. As a result, it is not uncommon that this process lasted over days. The warm solution is stored overnight in a refrigerator which turns into a gel and is warmed back into a solution the following day, prior to recommencing with the construction of these gelatin models. Thus, all equipment, such as beakers and syringes, that have been contact with gelatin solution, are washed and soaked overnight to deter the accumulation of gelatin clumps. It must be noted that these models have a lifetime in the order of a few weeks (if refrigerated regularly, while not in use) as gelatin is susceptible to microbial growth. The gelatin surface that shall be in direct contact with the ultrasound transducer is most prone to erosion and in the worst case scenario, fissures may appear in the models, which is further exacerbated after the model is cooled in the refrigerator. Once the model has been used or microbial growth appears, it is disposed off in the gel state.

The above process can be optimized further. For example, if an extremely accurate construction of models is desired, then the physical properties of the gelatin, such as mass density and speed of sound, as a function of gelatin concentration as well as temperature can be accounted for (Davis and Oakes 1922; Parker and Povey 2012). Moreover, the lifetime of these models can be increased further by additives that could prevent bacterial invasion, such as p-methyl and p-propyl benzoic acid. Alternative techniques can be developed as well, with totally different ingredients (Culjat et al. 2010). In fact, we also attempted to create similar models by suspending particles in ultrasound transmission gel (Aquasonic). An advantage of using ultrasound transmission gel over gelatin would be the lower absorption of sound. While the particles could be immobilized due to the high viscosity of the ultrasound transmission gel ($\approx$ 10^5^ times higher than water), the model is not stiff and is easily eroded upon contact with the transducer. Finally, the constructed gelatin models can be imaged in X-ray CT scanners to assess the construction and the true distribution of particles (for example, it is not uncommon to have more particles accumulate in the bulk rather than nearby the container walls).
